# Unravelling the genome of Holy basil: an “incomparable” “elixir of life” of traditional Indian medicine

**DOI:** 10.1186/s12864-015-1640-z

**Published:** 2015-05-28

**Authors:** Shubhra Rastogi, Alok Kalra, Vikrant Gupta, Feroz Khan, Raj Kishori Lal, Anil Kumar Tripathi, Sriram Parameswaran, Chellappa Gopalakrishnan, Gopalakrishna Ramaswamy, Ajit Kumar Shasany

**Affiliations:** Biotechnology Division, CSIR-Central Institute of Medicinal and Aromatic Plants, P.O. CIMAP, Lucknow, 226015 U.P. India; Microbial Technology Division, CSIR-Central Institute of Medicinal and Aromatic Plants, P.O. CIMAP, Lucknow, 226015 Uttar Pradesh India; Metabolic and Structural Biology Department, CSIR-Central Institute of Medicinal and Aromatic Plants, P.O. CIMAP, Lucknow, 226015 U.P. India; Genetics and Plant Breeding Division, CSIR-Central Institute of Medicinal and Aromatic Plants, P.O. CIMAP, Lucknow, 226015 U.P. India; Research and Development Unit, Genotypic Technology Private Limited, Bangalore, Karnataka 560094 India

**Keywords:** Chloroplast, Mitochondria, *Ocimum sanctum* L, Secondary metabolism, SSR’s Whole genome sequencing

## Abstract

**Background:**

*Ocimum sanctum* L. (*O. tenuiflorum*) family-Lamiaceae is an important component of Indian tradition of medicine as well as culture around the world, and hence is known as “Holy basil” in India. This plant is mentioned in the ancient texts of Ayurveda as an “elixir of life” (life saving) herb and worshipped for over 3000 years due to its healing properties. Although used in various ailments, validation of molecules for differential activities is yet to be fully analyzed, as about 80 % of the patents on this plant are on extracts or the plant parts, and mainly focussed on essential oil components. With a view to understand the full metabolic potential of this plant whole nuclear and chloroplast genomes were sequenced for the first time combining the sequence data from 4 libraries and three NGS platforms.

**Results:**

The saturated draft assembly of the genome was about 386 Mb, along with the plastid genome of 142,245 bp, turning out to be the smallest in Lamiaceae. In addition to SSR markers, 136 proteins were identified as homologous to five important plant genomes. Pathway analysis indicated an abundance of phenylpropanoids in *O. sanctum*. Phylogenetic analysis for chloroplast proteome placed *Salvia miltiorrhiza* as the nearest neighbor. Comparison of the chemical compounds and genes availability in *O. sanctum* and *S. miltiorrhiza* indicated the potential for the discovery of new active molecules.

**Conclusion:**

The genome sequence and annotation of *O. sanctum* provides new insights into the function of genes and the medicinal nature of the metabolites synthesized in this plant. This information is highly beneficial for mining biosynthetic pathways for important metabolites in related species.

**Electronic supplementary material:**

The online version of this article (doi:10.1186/s12864-015-1640-z) contains supplementary material, which is available to authorized users.

## Background

*Ocimum sanctum* L. (*O. tenuiflorum*) is an important sacred medicinal plant of India known as “holy basil”, *Thulasi*, *Vishnupriya,* and *Tulsi* [1] and worshipped for over more than 3000 years [2, 3]. This herb is popular in traditional medicine as “The Queen of Herbs,” “The Incomparable One,” and “The Mother Medicine of Nature” [4]. Being legendary sacred basil (Tulsi), is recognized [5, 6] not only for its sanctity, but forms an indispensible part of the traditional herbal medicine of East as discussed in Ayurvedic text of Charaka Samhita as well as Unani medicinal system. It is native to India and parts of northern and eastern Africa, Hainan Island, and Taiwan, and grows wild throughout India up to an altitude of 5900 ft (1800 m) in the Himalayas [7–9]. The leaf of the plant owes a stronger, somewhat pungent taste than other basils due to a sesquiterpenoid *beta-*caryophyllene, and a phenylpropanoid eugenol [10]. *O. sanctum* has been suggested to possess anti-fertility, anti-cancer, anti-diabetic, anti-fungal, anti-microbial, cardioprotective, analgesic, anti-spasmodic and adaptogenic actions [6]. The chemical composition of Tulsi is highly complex, containing many biologically active phytochemicals with variable proportions among varieties or even plants within the same field. The volatile oil of leaf [11] contains eugenol (1-hydroxy-2-methoxy-4-allylbenzene), euginal, urosolic acid [12], carvacrol, limatrol, caryophyllene, methyl carvicol while the seed volatile oil has fatty acids and sitosterol. In addition, the seed mucilage contains some levels of sugars and the anthocyans are present in green leaves [6]. The leaf volatiles (terpenes and phenylpropenes) are synthesized and sequestered in glandular hairs present on the leaves, also known as peltate trichomes, which are the characteristic of lamiaceae members [13, 14]. Two types of *O. sanctum* L. are used for cultivation: (i) plants with green leaves known as Sri/ Rama Tulsi & (ii) plants with purple leaves known as Krishna/ Shyama Tulsi [8]. Furthermore, the quantity of many of its constituents can be significantly altered by varying conditions used for growing; harvesting, processing and storage that are not yet well understood [15]. All of the varieties of *Ocimum* have unique and individual chemical compositions; but their medicinal properties are not yet explored completely. Despite huge importance of *Ocimum*, very little transcriptomic and genomic data of *Ocimum* sp. is available limiting studies on important phytochemical pathways. But comparative transcriptome analysis of *Ocimum* species (*O. sanctum* and *O. basilicum*) was recently reported [16]. This report correlated higher digital expression of phenylpropanoid/ terpenoid pathway genes of *O. basilicum* to higher essential oil content and chromosome number (*O. sanctum*, 2n = 16; and *O. basilicum*, 2n = 48). Also several cytochrome P450s (26) and transcription factor families (40) were identified which could be utilized to characterize genes related to secondary metabolism and its regulation.

Hence, there was a need to know about the genome of this plant to understand its metabolic potential, diversity, regulation and evolutionary implications. Here, we report the draft nuclear genome sequence of 386 Mb and the plastid of 142,245 bp sequenced with a composite next generation sequencing technologies. On the basis of assembly, 53,480 protein coding genes were identified. Gene model prediction revealed the similarity of *O. sanctum* genome to *Nicotiana tabacum* and *Solanum lycopersicum,* all sharing same sub-class (asterid)*.*

## Results and discussion

### Genome sequencing, assembly and validation

**Fig. 1:**
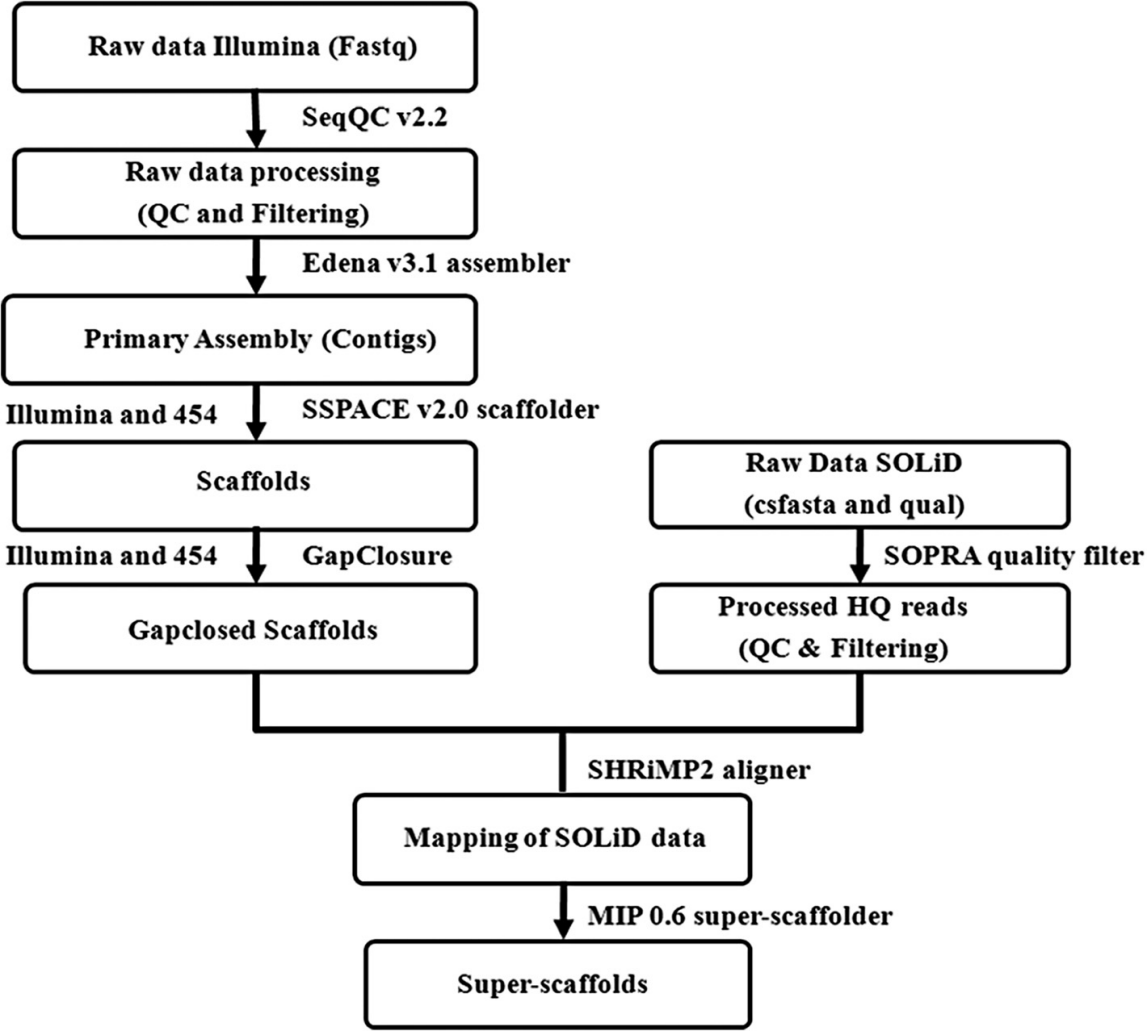
Process workflow of *Ocimum sanctum* whole genome sequencing and assembly

A whole-genome shotgun sequencing strategy by generating long and short paired-end reads, along with long reads and mate-pair libraries was applied to assemble the 386 Mb genome sequence of *O. sanctum.* The process workflow of the same has been provided in the Fig. 1. Two libraries (long and short reads) of Illumina HiSeq2000, one library of 454 GS FLX and one mate-pair library of SOLiD 5500XL were constructed. Illumina libraries were used to generate the contigs and Illumina paired-end data along with 454 GS FLX single end data were used for contig merging as a result of which scaffolds were generated. While Illumina generated 45.37 Gb data (Additional file 1), 454 sequencing resulted in 320.3 Mb (Additional file 2) data and SOLiD generated 12.68 Gb (Additional file 3) data representing approximately ~130-fold coverage of the predicted *O. sanctum* genome. With the help of the two Illumina library data (short insert and long insert) the assembly showed significant improvement in respect of N50. Long- and short-paired end reads from Illumina deep sequencing was used to assemble a total of 107,785 contigs into 22,776 scaffolds. Super-scaffolding was performed in order to merge the existing gap-closed scaffolds into super-scaffolds using relative orientation of SOLiD mate pair reads. Finally, 9059 super-scaffolds of maximum length upto 2,211,552 bp were generated (Table 1). Out of the total super-scaffolds generated, 4159 super-scaffolds are larger than or equal to 1 kb in length (Table 1). The N50 length of contigs, scaffolds and super-scaffolds was found to be 12,769 bp, 61,854 bp and 303,233 bp respectively (Table 1). The total length of the gaps in the assembled scaffolds was 26.11 Mb in a total of 3999 super-scaffolds. The total number of gaps present is 45,803 considering even the presence of a single N as one gap. The biggest gap identified was of 4906 bp in length. Mate-pair reads significantly closed ~60 % of gaps between scaffolds and five-fold change in N50 value and N90 value was also observed where N50 increased from 61,242 to 303,233 while N90 from 12,534 to 73,672. In order to validate the genome assembly, a total of 69,117 transcripts generated from our previous study [16] were mapped to the genome data and more than 95 % transcripts (66,891) showed 100 % coverage (Additional file 4).

**Table 1:** Assembly statistics of contigs and scaffolds generated using the three sequencing platforms Illumina HiSeq2000, 454 GS FLX and SOLiD 5500XL

Description	Contigs	Scaffolds	GapClosed scaffolds	Super-scaffolds
Contigs generated	107785	22776	22776	9059
Maximum Contig Length	115044	414711	411690	2211552
Minimum Contig Length	147	200	200	200
Average Contig Length	3454	16984	16629	44354
Total Contigs Length	372395755	386828951	378759759	401803260
Total Number of non-ATGC characters	0	17898452	2665702	26110056
Percentage of non-ATGC characters	0	4.627	0.704	6.498
Contigs > = 1 Kb	43174	14791	14769	4159
Contigs > = 10 Kb	11594	7544	7407	2357
N50 value	12769	61854	61242	303233
N90 value	2071	12742	12534	73672

### *De-novo* assembly of chloroplast and mitochondria genome data

**Fig. 2:**
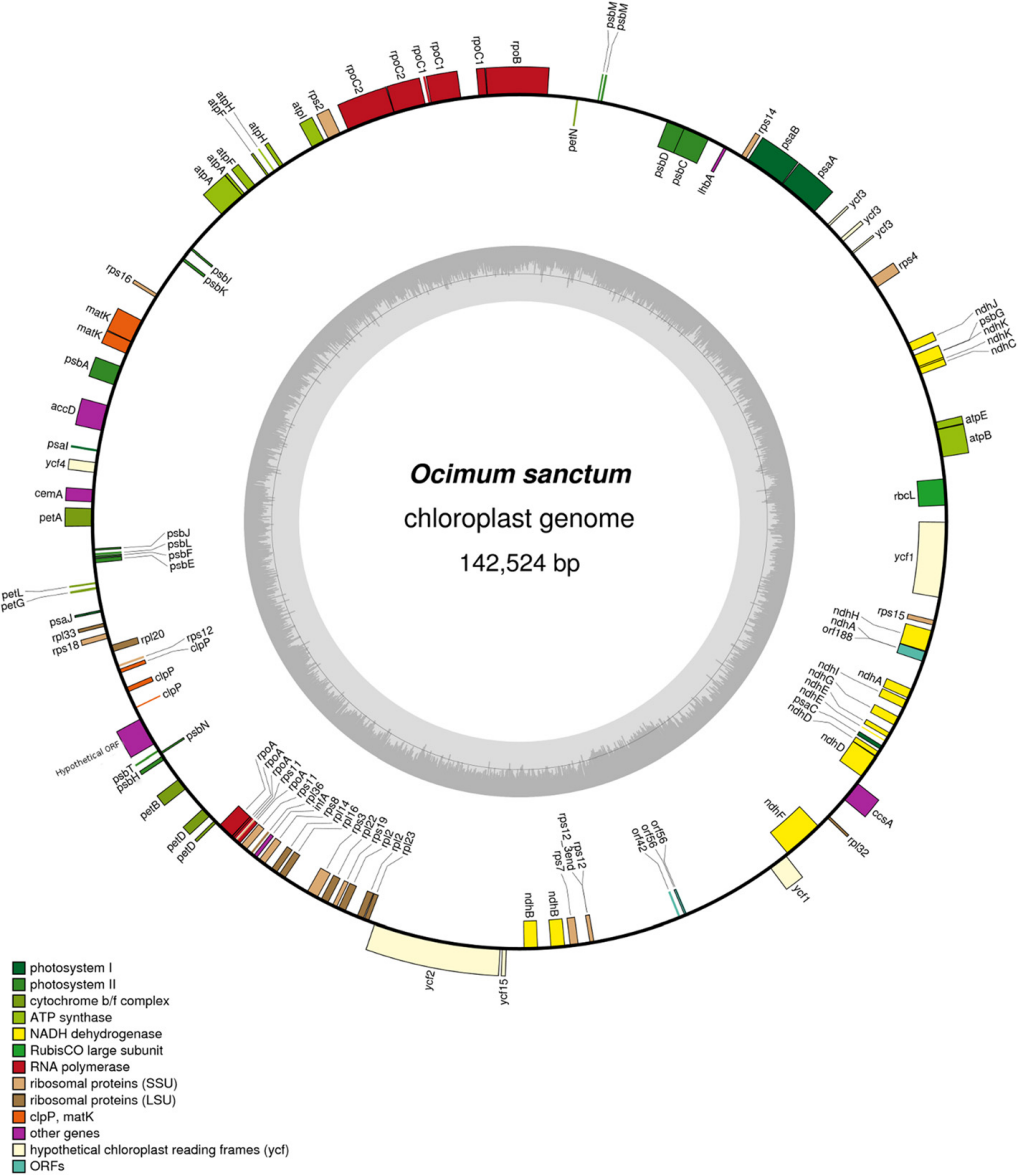
Gene map of the *Ocimum sanctum* chloroplast genome Genes drawn inside the circle are transcribed clockwise, and those outside are counterclockwise. Genes belonging to different functional groups are color-coded. The darker gray in the inner circle corresponds to GC content, while the lighter gray corresponds to AT content

The complete chloroplast (cp) genome of *O. sanctum* is 142,524 bp in length (Fig. 2). Recently, Qian et al [17] had reported the chloroplast genome of *Salvia miltiorrhiza* to be the smallest with the exception of *Epifagus virginiana* [18] cp genome of order lamiales. But this investigation revealed *O. sanctum* cp genome to be 8804 bp smaller than *S. miltiorrhiza* (member of the *Ocimum* family- lamiaceae) cp genome of length 151,328 bp. Hence *O. sanctum* cp genome is now reported as the smallest of the Lamiales cp genomes as it is ~8800 bp smaller than *E. virginiana* cp genome. The overall GC content of the *O. sanctum* cp genome is 36.2 %, which is similar to the other reported asterid cp genomes [17, 19–22]. The *O. sanctum* cp genome was found to code a total 158 genes, including 43 transfer RNA (tRNA) genes and four ribosomal RNA (rRNA) genes. The aligned reads of cp genome of *O. sanctum* to other angiosperms (referred in materials and methods section) were assembled into contigs for finally generating the scaffolds using all the Illumina data. Similar procedure was carried out for mitochondrial genome (Additional file 5 and 6) assembly except considering *S. miltiorrhiza* as the reference mitochondial genome. A total of 48 scaffolds from 140 contigs from cp genome, and 41 scaffolds from 124 contigs (Additional file 5 and 6) from the mitochondrial genome got generated.

### Genomic composition and SSR prediction

GC content is an important indicator of the genomic composition including evolution, gene structure (intron size and number), gene regulation and stability of DNA [23]. Average GC content of *O. sanctum* was 38.37 %. Earlier researchers have reported that across the broad phylogenetic sweep, genome size may be correlated with intron size [24–26], suggesting that some fraction of genome size evolution takes place within genes [27]. While performing the annotation of gene models, taking *N. tabacum* and *S. lycopersicum* as references, it was found that the percent genes containing introns from these plants were 55.5 % and 64.5 %, respectively (Additional file 7). It has been observed that introns and their positions are highly conserved during land plant evolution excluding conifers [28, 29].

Comparative studies had revealed that intron lengths and the abundance of mobile repetitive elements have a direct correlation with genome size, such that large genomes have longer introns and a higher proportion of mobile elements [30, 31]. Intron sizes in the genes of *O. sanctum* ranged from 5 bp to 8000 bp (Additional file 7). A reason for intron size variation among organisms may be due to inherent mutational processes generating insertions and deletions [24, 32–35]. It was also reported that low distribution of recombination regions leads to increased intron size [36, 37].

**Fig. 3:**
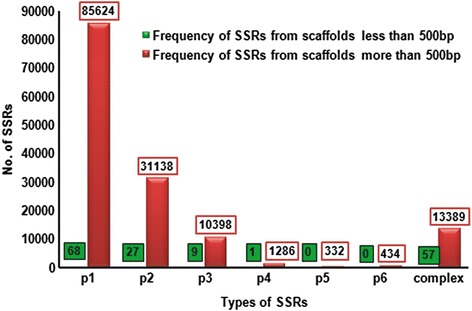
Frequency distribution of SSRs based on motif types. p1: Mono-nucleotide repeats; p2: Di-nucleotide repeats; p3: Tri-nucleotide repeats, p4: Tetra-nucleotide repeats; p5: Penta-nucleotide repeats; p6: Hexa-nucleotide repeats; Complex: no. of SSRs involved in compound formation

Among different classes of molecular markers, microsatellite or simple sequence repeat (SSR) markers are the most preferred for its array of applications in plant genetics and breeding due to their multi-allelic nature, reproducibility, co-dominant inheritance with high abundance and wide genome coverage [38, 39]. A total of 4827 sequences greater than 500 bp length were examined for SSR search out of which 2612 sequences were found having SSR repeats while 2364 sequences showed the presence of more than one SSR (Fig. 3). A sum of 142,601 SSRs were predicted, with the highest being mono-repeats (85,624) and (13,389) complex SSR’s. The sequences were checked for mono-repeats occurring at-least 10 times, di-repeats occurring at-least 6 times and tri/ tetra/ penta/ hexa-repeats occurring atleast 5 times. The SSR was classified as complex when two SSR’s were present within 100 bp distance of each other. On the other side, 1,166,753 sequences of less than 500 bp were identified, only 162 SSRs highest being mono-repeats (68) followed by complex SSR’s (57) (Fig. 3). Previously, we reported SSRs from the transcriptome of *O. sanctum* [16] but the SSRs identified were very few in comparison to the present report. Similarly, efforts were made by researchers towards the development of molecular markers in order to carry out genetic diversity studies on *Ocimum sp.* [40–42]. But, SSRs reported from the present study with a large data set would be helpful in providing insights to the plant breeders and geneticists for evaluation of desired genotypes with varied essential oil compositions and also for further development of new species of *Ocimum*.

A gene density of ~30 genes per 100 kb and ~20 genes per 100 kb was observed in *O. sanctum* gene model prediction taking *N. tabacum* (tobacco) and *S. lycopersicum* (tomato), respectively as references. Since *O. sanctum* is a small genome plant, the gene density is similar to that of *Arabidopsis thaliana* i.e., upto 38 genes per 100 kb [43]. Large genomes like barley and wheat show a gene density of about 5 genes per 23 kb [44] as it was suggested that the larger genomes would have accumulated non-coding sequences between the single-copy genes [45].

### Gene prediction and annotation

**Fig. 4:**
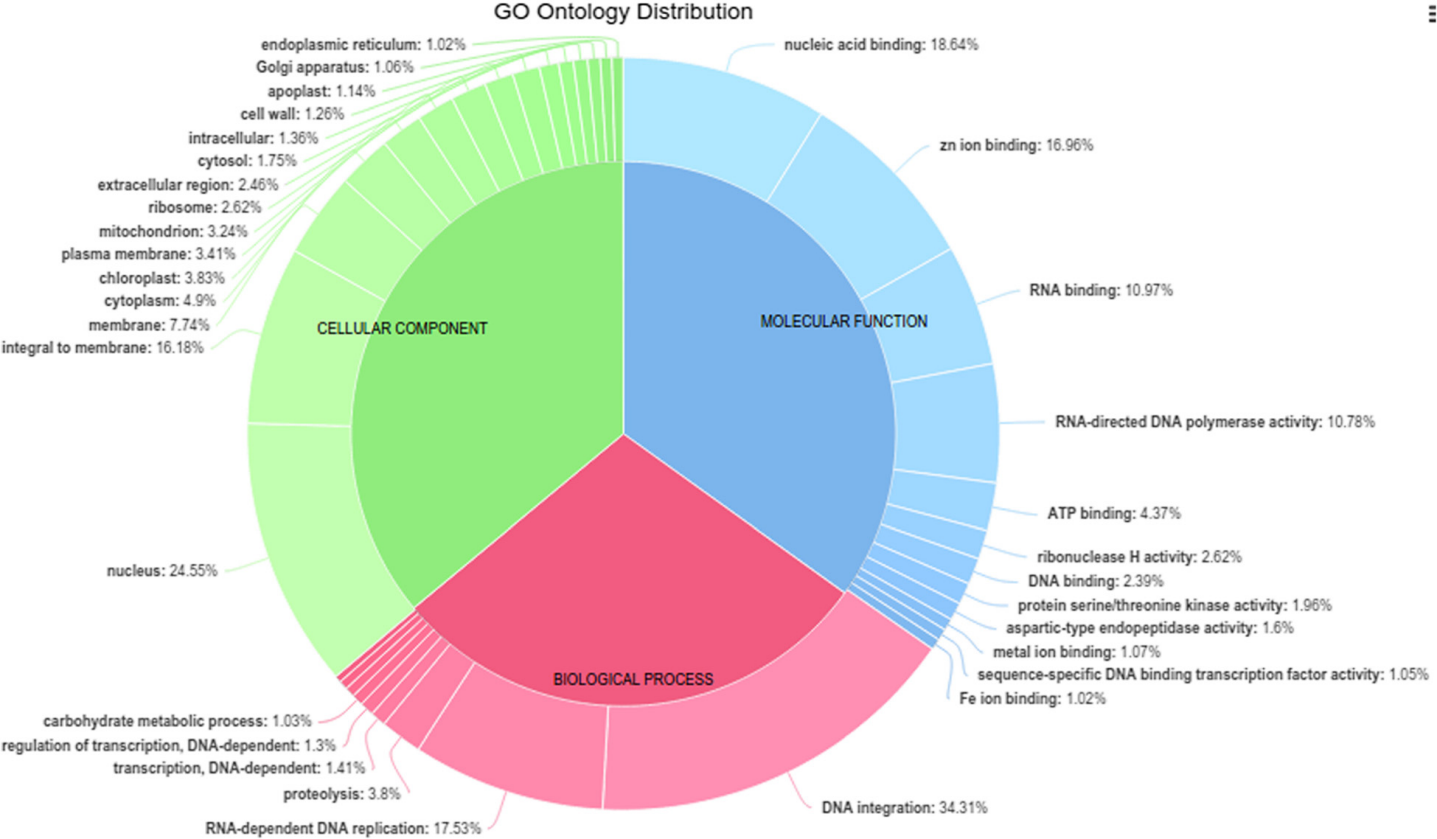
Pie-chart showing top 10 functional classes in each of the 3 categories of gene ontology classification. The three main categories are: biological process, cellular component and molecular function representing the assignment *O. sanctum* predicted proteins with BLAST matches in NR (non-redundant) protein sequences of *Arabidopsis* to each GO term

In order to assign putative functions to the predicted genes of *O. sanctum*, they were compared against the NR (non-redundant) protein sequences of *Arabidopsis*. The associated hits were searched for their respective GO. Based on sequence homology, 85,723 protein sequences were categorized into 31 functional groups under three main categories: biological processes (BP), cellular components (CC) and molecular functions (MF) (Fig. 4, Additional file 8). Genes were predicted from 22,776 scaffolds by mapping (BLASTP) the predicted proteins with UNIPROT with all Viridiplantae clade protein sequences. Out of 85,723 protein coding loci from 22,776 scaffolds, a total of 53,480 were annotated with UNIPROT (Additional file 9) but only 22,270 protein coding genes were found to be unique. On observing the plant species distribution of hits to UNIPROT database, maximum hits were from the plant *Genlisea aurea* (Additional file 10) which is one of the smallest known genome among higher plants [46]. The number of unique protein coding genes (22,270) in the *O. sanctum* genome, was in range as reported in potato, tomato [47], neem [48] and grapevine [49], having 35,004, 34,727, 20,000 and 30,434 protein coding genes, respectively.

*Ab initio* gene model prediction was performed on scaffold sequences utilizing minimal information from the nearest available species. Overall, 130,526 and 87,918 proteins were predicted using training sets of *Nicotiana tabacum* and *Solanum lycopersicum* respectively. A total of 65,935 proteins were common between the two predictions. Gene annotation of the predicted proteins with BLASTP resulted in annotation of 80,516 NR proteins. A set of 38,868 of these annotated proteins were common to the predictions from *N. tabacum* and *S. Lycopersicum*, respectively. The un-annotated predicted proteins were scanned with Pfam and another 18,940 proteins got annotated with a predicted domain signature. Database annotation of assembled scaffold sequences greater than 500 bp was carried out for matching with the EST/mRNA sequences available for *Ocimum* in the NCBI databases (Additional file 11). A total of 23,420 EST and 52 mRNA were queried, with a match to the assembled scaffolds for 21,984 of the EST/mRNA sequences at greater than 90 % sequence identity. Also *Arabidopsis* sequences (Additional file 12) from TAIR database and *N. tabacum* (Additional file 13) and *S. lycopersicum* (Additional file 14) sequences from NCBI were Blast- checked against the *O. sanctum* scaffolds with percent hitting scaffolds of 34.65 %, 4.9 % and 5.29 %, respectively. Database annotation of EST/mRNA from NCBI datasets identified the mitochondria and chloroplast expressed proteins. All of the 392 scaffolds identified were annotated to potentially map to these sequences (Additional file 15). Out of 392 scaffolds, 270 were redundant and only 122 were non-redundant. On the basis of annotation of chloroplast and mitochondria encoded proteins against TAIR database, it was found that out of 122 non-redundant scaffolds of *O. sanctum*, 95 were chloroplastic while remaining 27 were mitochondrial.

### Phylogenetic analysis

**Fig. 5:**
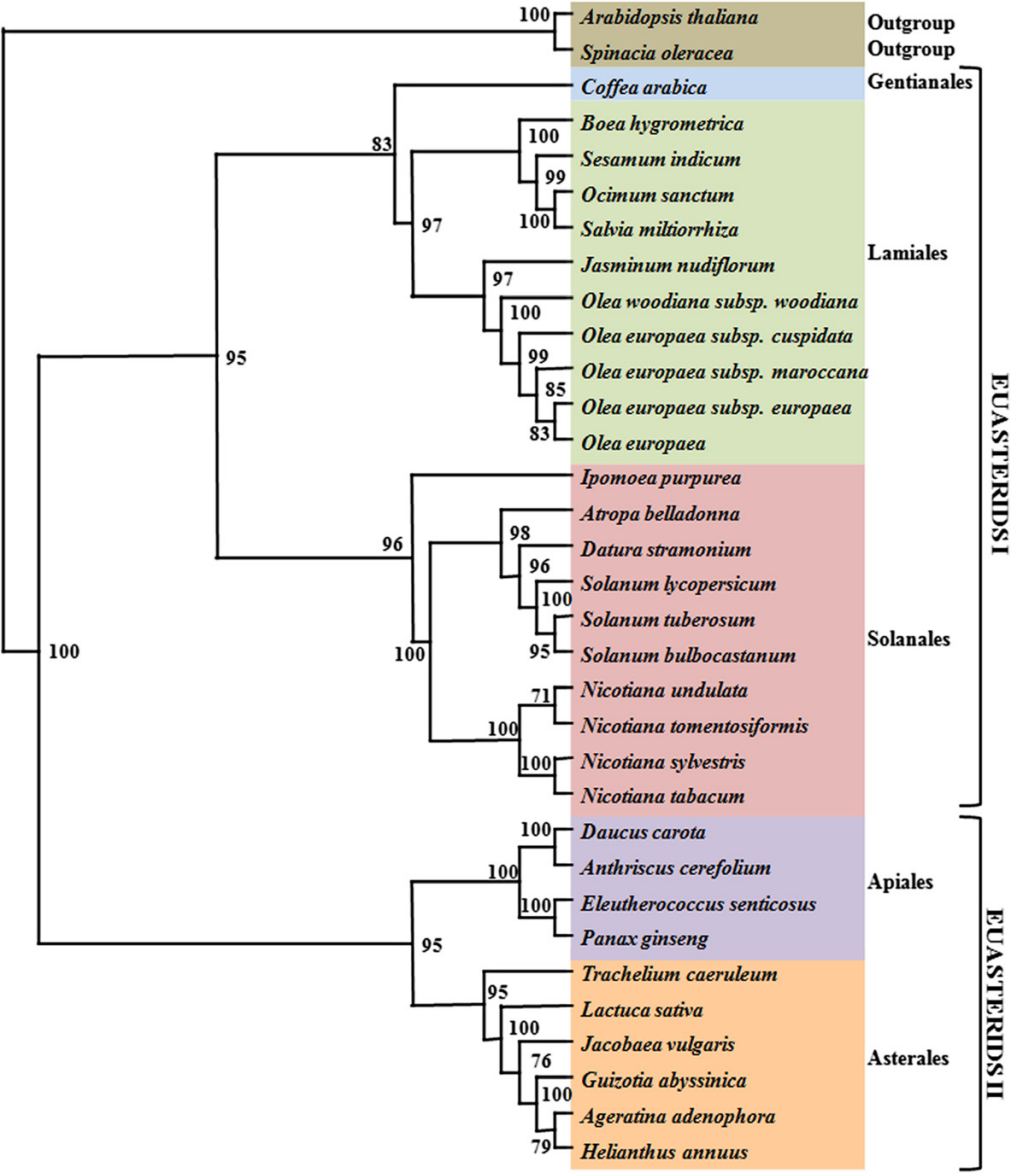
The phylogenetic tree of the asterid clade based on 63 protein-coding genes of chloroplast genome. Numbers above each node are bootstrap support values. *Spinacea oleracea* and *Arabidopsis thaliana* were set as outgroups

 To identify the phylogenetic position of *O. sanctum* within the asterid lineage, multiple sequence alignments was performed using 63 protein-coding genes (Additional file 16) commonly present in the 32 complete cp genomes representing 10 families within five orders of asterids including Apiaceae, Araliaceae, Asteraceae, Convolvulaceae, Gesneriaceae, Lamiaceae, Oleaceae, Pedaliaceae, Rubiaceae and Solanaceae (Additional file 17). Two additional eudicot cp genomes, *Spinacia olerace*a and *Arabidopsis thaliana*, were set as outgroups. A phylogenetic tree was generated using maximum parsimony and maximum likelihood method (Fig. 5). Bootstrap analysis showed that there were 25 out of 31 nodes with bootstrap values >95 %, and 14 of these had a bootstrap value of 100 %. The tree topologies formed two major clades, euasterids I and II. The results strongly supported the position of *Ocimum sanctum* and *Salvia miltiorrhiza* with 100 % bootstrap from the same family lamiaceae as the sister of the closely related species *Sesamum indicum* and *Boea hygrometrica* in the order Lamiales. As the chloroplast genome is considered to be free from evolutionary processes, like gene duplication/ deletion, intensive evolution and pseudogene formation, which are characteristically frequent among nuclear genes, this was included in the phylogenetic analysis [50]. Slow rate of sequence evolution in chloroplast DNA is appropriate to include cp genome in phylogenetic studies of the highly cross pollinated plant like *O. sanctum* [51].

### Pathway identification

**Fig. 6:**
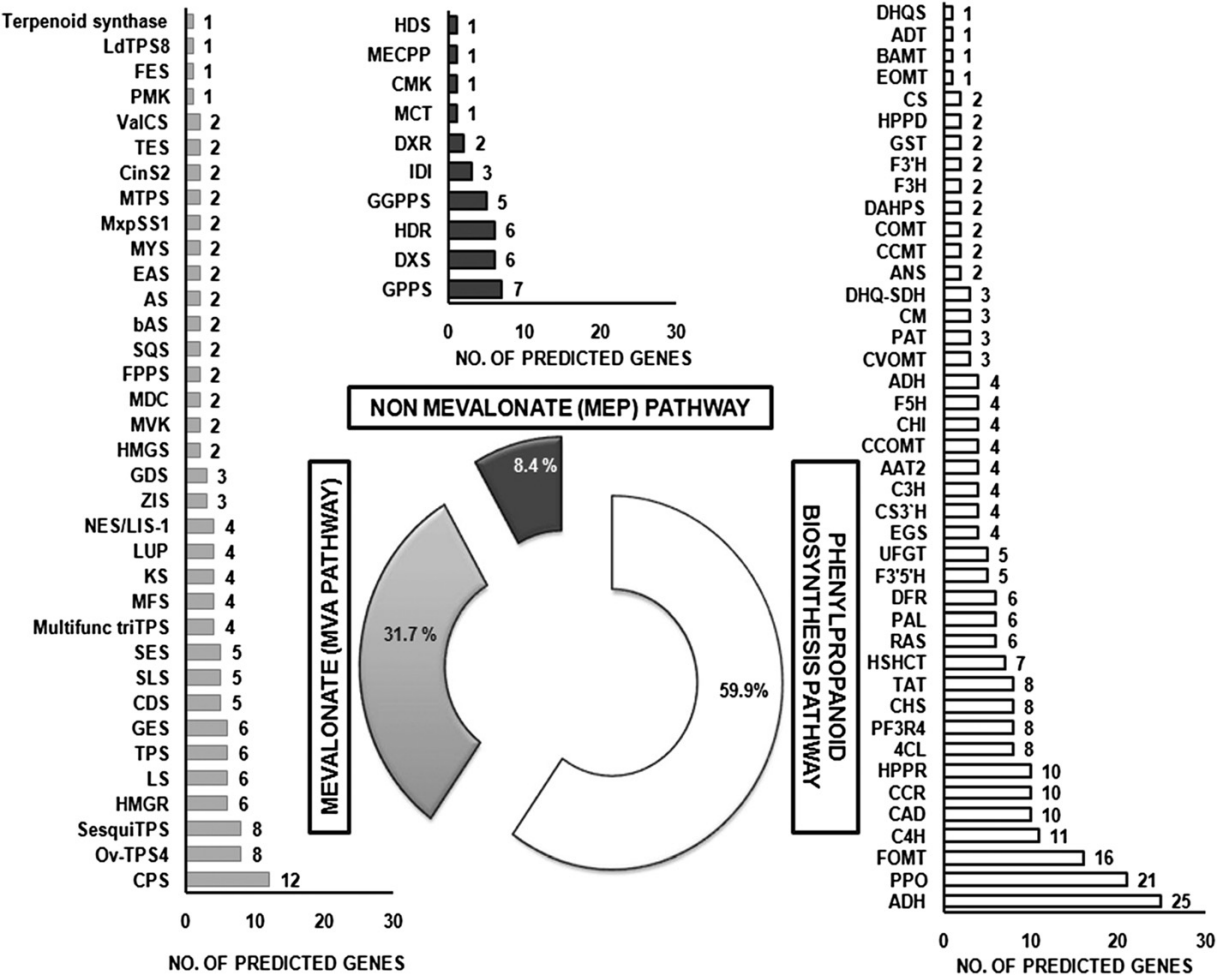
Abundance of phenylpropanoid, mevalonate and non- mevalonate pathway genes as per the annotation of predicted genes against all Viridiplantae clade genes in Uniprot. [Abbreviations used- Alcohol dehydrogenase (ADH); Polyphenol oxidase (PPO); Flavonoid O-methyltransferase (FOMT); Cinnamate 4-hydroxylase (C4H); Cinnamyl alcohol dehydrogenase (CAD); Cinnamoyl-CoA reductase (CCR); Hydroxyphenylpyruvate reductase (HPPR); 4-Coumarate:coenzyme A ligase (4CL); Anthocyanidin 3-O-glucoside 5-O-glucosyltransferase (PF3R4); Chalcone synthase (CHS); Tyrosine aminotransferase (TAT); Hydroxycinnamoyl transferase (HSHCT); Rosmarinic acid synthase (RAS); Phenylalanine ammonia-lyase (PAL); Dihydroflavonol 4-reductase (DFR); Flavonoid 3’ 5’-hydroxylase (F3’5’H); UDP-glucose: flavonoid 7-O-glucosyltransferase (UFGT); Eugenol synthase 1 (EGS); *p*-Coumaroyl shikimate 3’-hydroxylase (CS3’H); *p*-Coumarate 3-hydroxylase (C3H); Alcohol acyltransferase (AAT2); Caffeoyl CoA *O*-methyltransferase (CCOMT); Chalcone isomerase (CHI); Ferulate 5-hydroxylase (F5H); Arogenate dehydrogenase (ADH); Chavicol O-methyltransferase (CVOMT); Prephenate aminotransferase (PAT); Chorismate mutase (CM); Dehydroquinate dehydratase/ shikimate dehydrogenase (DHQ-SDH); Anthocyanidin synthase (ANS); Cinnamate/p-coumarate carboxyl methyltransferase (CCMT); Caffeic acid 3-*O*-methyltransferase (COMT); 3-deoxy-D-arabino-heptulosonate 7-phosphate synthase (DAHPS); Flavanone 3-hydroxylase (F3H); Flavonoid 3’-hydroxylase (F3’H); Glutathione S-transferase (GST); 4-Hydroxyphenylpyruvate dioxygenase (HPPD); Chorismate synthase (CS); Eugenol *O*-methyltransferase (EOMT); Benzoate carboxyl methyltransferase (BAMT); Arogenate dehydratase (ADT); 3-dehydroquinate synthase (DHQS); Copalyl diphosphate synthase(CPS); Bicyclogermacrene synthase (*Ov*-TPS4); Sesquiterpene synthase (SesquiTPS); 3-hydroxy-3-methylglutaryl-coenzyme A reductase (HMGR); (R)-limonene synthase (LS); Terpene synthase (TPS); Geraniol synthase (GES); Gamma-cadinene synthase (CDS); Secologanin synthase (SLS); Selinene synthase (SES); Multifunctional triterpene synthase (Multifunc triTPS); (+)-menthofuran synthase (MFS); Ent-kaurene synthase (KS); Lupeol synthase(LUP); Nerolidol/linalool synthase (NES/LIS-1); Alpha-zingiberene synthase (ZIS); Germacrene D synthase (GDS); 3-hydroxy-3-methylglutaryl coenzyme A synthase(HMGS); Mevalonate kinase (MVK); Mevalonate diphosphate decarboxylase (MDC); Farnesyl diphosphate synthase(FPPS); Squalene synthase (SQS); *Beta*-amyrin synthase (bAS); Mixed amyrin synthase (AS); 5-epi-aristolochene synthase (EAS); *Beta*-myrcene synthase (MYS); *Cis*-muuroladiene synthase (MxpSS1); Monoterpene synthases (MTPS); Cineole synthase (CinS2); Terpinolene synthase (TES); Valencene synthase (ValCS); 5-phosphomevalonate kinase (PMK); (-)-endo-fenchol synthase (FES); (+)-epi-alpha-bisabolol synthase (*Ld*TPS8); Tricyclene synthase 0e23 /(E)-beta-ocimene synthase 0e23/ Terpenoid synthase 0e23) (Terpenoid synthase); Geranyl diphosphate synthase (GPPS); 1-deoxy-D-xylulose 5-phosphate synthase (DXS); 4-hydroxy-3-methylbut-2-enyl diphosphate reductase (HDR); Geranylgeranyl diphosphate synthase (GGPPS); Isopentenyl diphosphate isomerise (IDI); 1-deoxy-D-xylulose-5-phosphate reductoisomerase (DXR); 2-C-methyl-D-erythritol 4-phosphate cytidylyltransferase (MCT); 4-diphosphocytidyl-2-C-methyl-D-erythritol kinase (CMK); 2-C-methyl-D-erythritol 2,4-cyclodiphosphate synthase (MECPP); 4-hydroxy-3-methylbut-2-enyl diphosphate synthase (HDS)]

To identify the biological pathways functional in *O. sanctum* 85,723 protein sequences from scaffolds were mapped to the reference canonical pathways in KEGG taking *Arabidopsis thaliana* and *Oryza sativa* as reference organisms, out of which only 6328 proteins got predicted in KAAS (Additional file 18). All transcripts were classified mainly under five categories: metabolism, cellular processes, genetic information processing, environmental information processing and others. Highest numbers of sequences were related to metabolism. Maximum percentage of the sequences fell under the category of phenylpropanoid biosynthesis. *O. sanctum* is good source of phenylpropene- eugenol and is one of the compounds which attributes to its medicinal property [52, 53]. Precursor molecules for phenylpropanoid biosynthesis are derived from the shikimate pathway while terpenoid biosynthesis utilizes isoprenoid precursors from cytosolic MVA (mevalonate) as well as plastidial MEP pathways (2-Cmethyl-D-erythritol 4-phosphate/1-deoxy-D-xylulose 5-phosphate/non-mevalonate pathways) [16]. On sorting 53,480 protein coding genes of *O. sanctum* annotated from UNIPROT for phenylpropanoid (Fig. 6) and terpenoid (MEP and MVA) pathway genes (Fig. 6) it was found that the highest number of phenylpropanoid pathway genes were identified as compared to the terpenes. Since the *O. sanctum* variety used in the present study is high-yielding, eugenol-rich, oil producing variety [54] with 83 % eugenol in the oil, it correlates with the presence of higher number of phenylpropanoid pathway genes. Interestingly, highest number transcripts of copalyl diphosphate synthase (CPS) were present among the mevalonate pathway genes (Fig. 6). This enzyme participates in gibberellin biosynthesis [55].

Not only gibberellins, but a wide range of secondary metabolites, including terpenes and alkaloids, are also derived either from *ent*-copalyl pyrophosphate itself or from *ent*-kaurene or *ent*-kaurenoic acid, the next two intermediates in the metabolic pathway to gibberellins. Knowledge of these secondary metabolic pathways is very much limited as compared to gibberellin biosynthetic pathway, and is often little more than a speculation [56]. Further functional characterization studies for copalyl diphosphate synthase may help in proving the possibility of CPS involvement in terpene and alkaloid biosynthesis.

### Medicinal nature of *O. sanctum*

In this analysis *O. sanctum* cp genome was observed to be evolutionarily nearest to *S. miltiorrhiza*. In the absence of complete genome sequence data (unfinished draft genome) of *S. Miltiorrhiza*, the chloroplast genome comparison analysis was taken into account. Both the plants are used widely in two different traditional medicine systems (Indian and Chinese, respectively), and hence may be implicated for similar molecules, activities *vis a vis* the genes biosynthesizing metabolites. In addition, both plants have chromosome number described to be 2n = 16 [16, 57]. The active ingredients in *S. miltiorrhiza* are both hydrophilic (phenolic acids like rosmarinic acid, salvianolic acid B, lithospermic acid and dihydroxyphenyllactic acid) and lipophilic diterpene components (tanshinones, including structurally related tanshinone I, tanshinone IIA, cryptotanshinone, and dihydrotanshinone I) [58]. These molecules are responsible for a wide array of activities like anti-bacterial, anti-oxidative and anti-viral to hepatoprotective activities. The chemical composition of Tulsi is highly complex, and the important are triterpene like urosolic acid (cardioprotective effect), phenolics like rosmarinic acid, apigenin, cirsimaritin, isothymusin and isothymonin (exhibiting antioxidant and anti-inflammatory activities), and important aroma components like 1, 8 cineole, linalool, methyl chavicol (estragole) and eugenol [16]. Phenolic acid compounds production by hairy root culture have been reported in both *O. basilicum* and *S. miltiorrhiza* [59]. In addition, the vast literature indicates phenylpropanoid derivatives in these two plants are responsible for a range of major activities. In this investigation also we could observe the dominance of phenylpropanoid pathway genes. The highest number of sequences among the mevalonate pathway genes in *O. sanctum* are observed to be homologous to copalyl diphosphate synthases (CPS), that are involved in the biosynthesis of an important bioactive diterpene tanshinone in *S. miltiorrhiza* [58]. As *O. sanctum* is traditionally used for many aliments and the compounds of this plant are not fully investigated, the possibility exists for the discovery of tanshinone like compounds and other novel diterpenes.

## Conclusion

The genome of Holy basil, assembled *de novo* in this study, presents the smallest nuclear genome in the family Lamiaceae and smallest cp genome in the order Lamiales. Phylogenetically, *S. miltiorrhiza* is most similar to *O. sanctum* with a reported genome size of approximately ~600 Mb [17]. Although, both *S. miltiorrhiza* and *O. sanctum* predominantly produce phenylpropanoids and both have the identical diploid number of chromosome (2n = 16), the genome size of *O. sanctum* is little more than half of the genome size of *S. miltiorrhiza*. Hence, *O. sanctum* genome (386 Mb) seems to be quite compact with relatively less repeat sequences, even though it falls in the identical phylogenetic clade. In contrast to the genome sizes of the plants used in the gene model prediction like *Solanum lycopersicum* (~900 Mb) and *Nicotiana tabacum* (~4567 Mb), *O. sanctum* genome (~386 Mb) falls in the category of the plants with small genome and is just 1.5 times that of the model plant *Arabidopsis thaliana* (~135 Mb) while approximately same size as that of *Oryza sativa* (~420 Mb) [47, 60, 43, 61].

Besides the saturated genome sequence, this investigation also provides an assembled chloroplast genome, showing highest similarity to that of *S. miltiorrhiza*, an important medicinal plant of traditional Chinese medicine. Both the plants are rich in phenylpropanoids and their derivatives, and many of these are implicated for different therapeutic activities. The presence of large number of homologs of copalyl diphosphate synthases (CPS) in *O. sanctum* genome indicates the possibility of finding newer diterpenes having potential bioactivity not implicated so far. Genomic information generated in this investigation not only is an important resource for evolutionary studies it will also catalyze modern genetic research by augmenting the data available for plant comparative genomics. This will also accelerate identification of important secondary metabolite-synthesizing genes, not identified yet from this medicinal and aromatic plant. Specific pathway related genes identified or mined in this genome could be used for the production of secondary metabolites following synthetic biology approaches. Genetic markers can be developed based on these genome sequences for studies involving genetic map construction, positional cloning, strain identification and marker-assisted selection. These molecular tools and genomic resources will accelerate molecular breeding and ultimately Holy basil’s utility in medical community.

## Methods

### Plant material, DNA preparation

Leaf tissues of *O. sanctum* L. (variety CIM Ayu) were collected from the experimental farm at the CSIR-Central Institute of Medicinal and Aromatic Plants. High molecular weight genomic DNA isolated (Plant DNA extraction kit, Qiagen) from the leaves of *O. sanctum* was analyzed for its concentration and integrity. This DNA was then used for a whole-genome shotgun and mate-pair library preparation.

### Library preparation methods

#### Long and short shot gun library construction

Long and short insert libraries for whole genome sequencing were constructed as per Illumina TruSeq DNA library (TruSeq DNA Sample Preparation Guide, Part No. 15005180 Rev. A, Nov 2010). 2 microgram of genomic DNA was used to prepare the DNA library acoustic shearing (Covaris Inc., USA) to a fragment distribution ranging between 150 to 600 bp and purified (Agencourt Ampure XP SPRI beads, Beckman Coulter, Inc.). Fragment distribution was analyzed (high sensitivity bioanalyzer chip, Agilent Technologies), finally purified (Agencourt Ampure XP SPRI beads) and quantified (Qubit fluorometer, Invitrogen as well as a high sensitivity bioanalyzer Chip, Agilent Technologies). The library shows a peak at the range of 300-400bp for short insert and 500-600bp for long insert libraries, respectively. Finally the libraries prepared were found suitable for 100bp paired end sequencing on Illumina.

#### Long reads 454 GS FLX library library construction

454 GS FLX library was constructed according to the Roche rapid library preparation method manual (GS FLX+ Series—XL+, May 2011). Briefly ~1ug genomic DNA was fragmented (using a nebulizer), purified (Minelute PCR purification kit, Qiagen) and end-repaired followed by adapter ligation. The prepared library was validated for quality (high sensitivity bioanalyzer chip, Agilent Technologies) which showed an expected peak range of 1.4–1.8 kb.

#### Mate-pair library construction

Mate pair libraries were generated as per the SOLiD Mate Pair Library preparation protocol. 23ug genomic DNA was sheared (ultra-sonicator, Covaris, USA) and analyzed for the size distribution (high sensitivity bioanalyzer chip, Agilent Technologies) also verified on 2 % E-gel. Next step was end-repairing of the fragments ranging from 2.5 to 3.5kb (resolved on 0.6 % agarose) followed by MPR-MPL adaptor ligation. Further, nick-translation was performed on circularized adaptor ligated DNA digested with T7 endonuclease I followed by S1 Nuclease enzymes. These products were 3’ adenylation by P1-T and P2-T, and captured using streptavidin beads (Invtirogen). Adaptor ligated sample was amplified with 18 cycles of PCR and size selected in the range of 250bp to 350bp using E-Gel (Invitrogen).

### Sequencing of shot-gun and mate-pair libraries and Genome assembly

Long and short insert libraries, were sequence on Hiseq2000 (Illumina) using 100 base paired end chemistry. Long single end reads were generated using Roche 454 (Roche) and mate-pair libraries were run on SOLiD 5500XL (Life technologies). Illumina generated 224,617,107 paired end reads (45.37 Gb data), 454 sequencing resulted in 643,134 single end reads (320.3Mb data) while SOLiD generated 126,824,255 mate pair reads (12.68 Gb data)

Long and short reads paired-end read data (HiSeq2000) of 449,234,214 (449 million) reads with high quality ( > = Q30) were assembled with Edena v3.1 [62]. Edena was used with default parameters, i.e. minimum overlap size being 50 and coverage cutoff, 4. Total genome coverage from the long and short insert paired-end reads was ~18.25X and ~82.55X (Additional file 1), respectively. 643,134 long single end 454 reads, processed for quality filtering with Phred score > =Q20 having a genome coverage of ~0.71X were then used for contig extension using SSPACE-2.03 [63]. SSPACE was used with these parameters: (i) minimum number of overlapping bases with the seed: 45, (ii) minimum overlap required between contigs to merge adjacent contigs in a scaffold:50, (iii) minimum number of read pairs to compute scaffold: 5 and contig extension switched on (iv) minimum number of reads needed to call a base during an extension: 20 and, (v) maximum number of allowed gaps during mapping with Bowtie: 1. Scaffolds thus generated do consisted of uncalled bases (Ns). Gap filling of these inter-scaffold Ns with nucleotides was carried out using GapClosure tool [64]. 252 million reads were generated using SOLiD showed ~30X coverage on the genome. SOLiD reads, with mean quality of Q20, and reads that have any uncalled bases (Ns) were filtered using SOPRA v1.4.6 [65] tool. Super-scaffolding was performed in order to merge the existing gap-closed scaffolds into super-scaffolds using relative orientation of SOLiD mate pair reads. Super-scaffolding using MIP-scaffolder [66] requires F3 and R3 reads to be mapped on preassembled scaffolds. This was achieved using SHRiMP2 [67] tool, which aligns reads in colorspace format.

### Gene prediction and annotation

*Ab initio* Gene model prediction was performed on scaffold sequences greater than 500bp using gene prediction software AUGUSTUS v2.5.5 [68]. Parameters from *N. tabacum* and *S. lycopersicum* species which share the same sub-class (asterid) with *Ocimum sanctum* were applied as training sets. Gene annotation of predicted proteins was done by matching to NCBI Non Redundant database using BLASTP (ncbi-blastv2.2.26+) [69]. Domain prediction for unannotated proteins was performed against Pfam (release 27) HMM signatures [70] using Pfam-A set with HMMSCAN option in HMMER 3.0 [71] at default parameters. Further scaffold sequences greater than 500bp in length were matched for match to EST/mRNA sequences available for *Ocimum* in the NCBI databases. *Arabidopsis* sequences from TAIR database were also BLAST checked against the *Ocimum* scaffolds (greater than 500bp). *Nicotiana* and *Solanum* EST’s from NCBI database were retrieved and matched against the assembled scaffolds which had length greater than 500bp.

### Comparative genomics and SSR prediction

The comparison of scaffolds with the *Ocimum* sequences was carried out using blat- Standalone BLAT v. 34x12 [72] fast sequence search command line tool. A total of 23,368 EST and 52 mRNA were queried, with a match to the assembled scaffolds for 21,984 of the EST/mRNA sequences at greater than 90 % sequence identity. *Arabidopsis* sequences from TAIR as well as *N. tabacum* and *S. lycopersicum* EST’s from NCBI database were also blast checked against the *O. sanctum* scaffolds (greater than 500bp). Apart from the database annotation of the assembled scaffolds these were also queried for intron length, intron distribution and gene density determination using AUGUSTUS v2.5.5 [68] with *N. tabacum* and *S. lycopersicum* as references.

Scaffold sequences of length less than 500bp as well as greater than 500bp were separately checked for simple sequence repeats (SSRs) using MISA tool (http://pgrc.ipk-gatersleben.de/misa/). The sequences were checked for mono-repeats occurring at-least 10 times, di-repeats occurring at-least 6 times and tri/tetra/penta/hexa-repeats occuring atleast 5 times.

### Annotation and *de-novo* assembly of chloroplast and mitochondrial genome data

Processed short reads paired-end read data of 72,912,212 (72.91 million) reads were aligned using BOWTIE2-2.1.0 [73] to “*Liquidambar formosana* (Accession no. KC588388.1), *Nandina domestica* (Accession no. DQ923117.1), *Arabidopsis thaliana* (Accession no. NC_000932), *Citrus sinensis* (Accession no. NC_008334), *Cucumis sativus* (Accession no. NC_007144), *Gossypium hirsutum* (Accession no. NC_007944), *Helianthus annuus* (Accession no. NC_007977), *Nerium oleander* (Accession no. KJ953906.1), *Oenothera biennis* (Accession no. NC_010361), *Platanus occidentalis* (Accession no. NC_008335), *Populus trichocarpa* (Accession no. NC_009143), *Spinacia oleracea* (Accession no. NC_002202), *Ximenia americana* (Accession no. HQ664594.1), *Ilex cornuta* (Accession no. HQ664579.1)*, Dillenia indica* (Accession no. HQ664593.1)*, Oxalis latifolia* (Accession no. HQ664602.1)*, Plumbago auriculata* (Accession no. HQ664581.1)*, Staphylea colchica* (Accession no. HQ664600.1)*, Lonicera japonica* (Accession no. HQ664582.1)*, Antirrhinum majus* (Accession no. HQ664592.1)*, Cornus florida* (Accession no. HQ664596.1)*, Ficus sp.* (Accession no. HQ664605.1) chloroplast genomes. Database annotation of EST/mRNA from NCBI datasets identified the mitochondria and chloroplast expressed proteins. These 122 scaffolds were annotated to potentially map to these sequences. The aligned reads were assembled using SPAdes-3.1.0 [74]. The assembled contigs were scaffolded using SSPACE-2.0 using all the four libraries Illumina data. Saffolds were gapclosed using Gapcloser-1.6. OrganellarGenomeDRAW (OGDRAW) was used for generating graphical maps of plastid genomes [75].

Similar procedure carried for mitochondria assembly except chloroplast genomes *Salvia miltiorrhiza* mitochondria genome used as reference and scaffolding and gapclosing was done using MIP-Scaffolder [66] using SOLiD data. Chloroplast Scaffolds greater than 10kb were filtered, ordered and joined with 2 N’s though using *Salvia miltiorrhiza* chloroplast genome. Annotation was carried from draft genome using DOGMA tool [76].

### Sequence divergence and phylogenetic analysis

The 32 complete cp sequences representing the asterid lineage of angiosperms were downloaded from NCBI Organelle Genome Resources database (Additional file 17). The 63 protein-coding gene sequences were aligned using the Clustal algorithm [77]. For the phylogenetic analysis, a set of 63 protein-coding genes commonly present in the 31 analyzed genomes was used. Maximum parsimony (MP) and Maximum likelihood (ML) analysis was performed for the phylogenetic analysis and the tree was generated using MEGA 6.0 [78] software. In the analysis *Spinacia oleracea* and *Arabidopsis thaliana* were set as outgroups.

### Genome annotation and pathway identification

85,723 protein coding sequences were blasted against NR proteins GO (Gene Ontology) terms were assigned for each protein based on the GO terms annotated to its corresponding homologue in the NR database. Each annotated sequence may have more than one GO term, assigned either for different GO categories (Biological Process, Molecular Function and Cellular Component) or in the same category [79].

Nucleotide sequences of the predicted proteins from scaffolds were retrieved (BEDTools-Version-2.13.1) [80] and mapped to KAAS [81] server to match pathway datasets from curated model species. Homology driven match of KO ID’s to best hits was done with default parameters. Match to model dicot and moncot plants *Arabidopsis* and *Oryza* were applied for pathway annotation.

### Data access

Genomic data generated by all the three platforms of *O. sanctum* whole project are available at NCBI under accession numbers SRX760129, SRR1653607 (Illumina); SRX760132, SRR1653610 (454_GS_FLX) and SRX761338, SRR1654829 (SOLiD). The data was submitted by SRA submission portal with submissionID, SUB745374 and BioProject ID, PRJNA267195.

### Availability of supporting data

The data sets supporting the results of this article are included within the article (and its additional files).

## Additional files

Additional file 1:
**Illumina libraries statistics.**
Additional file 2:
**Read length distribution chart for 454 reads.**
Additional file 3:
**SOLiD colorspace reads statistics.**
Additional file 4:
**Statistics of transcriptome and whole genome assembly of** ***O. sanctum.***
Additional file 5:
**QC statistics of chloroplast de novo assembly at each step.**
Additional file 6:
**QC statistics of mitochondrial de novo assembly at each step.**
Additional file 7:
***Ocimum sanctum*** **in comparison to** ***Nicotiana tabacum*** **and** ***Solanum lycopersicum.***
Additional file 8:
**Gene ontology classification.** The results are summarized in three main categories: biological process, cellular component and molecular function. Additional file 9:
**Annotation of predicted genes against all Viridiplantae clade genes in UNIPROT.**
Additional file 10:
**Top 10 Species distribution of hits to UNIPROT database.**
Additional file 11:
***Ocimum*** **EST blat check against** ***O. sanctum*** **scaffolds.**
Additional file 12:
***Arabidopsis*** **TAIR sequences blast check against** ***O. sanctum*** **scaffolds.**
Additional file 13:
***Nicotiana tabacum*** **EST blast check against** ***O. sanctum*** **scaffolds.**
Additional file 14:
***Solanum lycopersicum*** **EST blast check against** ***O. sanctum*** **scaffolds.**
Additional file 15
**Annotation of Mitochondrial/Chloroplast scaffolds based on the TAIR database.** ChrC_ChrM are the chloroplast and mitochondria sequences containing scaffolds based on TAIR ID. Additional file 16:
**List of 63 protein coding genes of cp genomes used in the phylogenetic analysis.**
Additional file 17:
**The list of accession numbers of the chloroplast genome sequences used in this study.**
Additional file 18:
**Pathways associated with predicted proteins from scaffolds of** ***O. sanctum*** **as predicted by KAAS server taking Arabidopsis and rice as reference organisms.**

## Abbreviations

Mb: Megabasepairs
bp: Basepairs
kb: Kilobasepairs
SOLiD: Sequencing by oligonucleotide ligation and detection
SSRs: Simple sequence repeats
cp: Chloroplast
GO: Gene ontology
BP: Biological process
CC: Cellular component
MF: Molecular function
UNIPROT: Universal protein resource database
DNA: Deoxyribonucleic acid
tRNA: Transfer ribonucleic acid
rRNA: Ribosomal ribonucleic acid
mRNA: Messenger ribonucleic acid
EST: Expressed sequence tag
NR: Non-redundant
NCBI: National Center for Biotechnology Information
TAIR: The Arabidopsis Information Resource
BLAST: Basic Local Alignment Search Tool
KEGG: Kyoto Encyclopedia of Genes and Genomes
KAAS: KEGG Automatic Annotation Server
SRA: Sequence Read Archive
