# Supplementary material for: Unravelling the genome of Holy basil: an “incomparable” “elixir of life” of traditional Indian medicine
Source: BMC Genomics. 2015 May 28;16(1):413. doi: 10.1186/s12864-015-1640-z (PMC4445982; doi:10.1186/s12864-015-1640-z)
Supplement: Additional file 1: — Illumina libraries statistics. [file 12864_2015_1640_MOESM1_ESM.pdf]

Additional File 1. Illumina libraries statistics

Library name	No. of reads	Total bases	Coverage (assuming 450Mb genome)
*O. sanctum*_Illumina_Long_insert_7	62217990 (62.2 million)	6284016990	13.96x
*O. sanctum*_Illumina_Long_insert	19134136 (19.1 million)	1932547736	4.29x
*O. sanctum*_Illumina_Short_insert_4	294235858 (294 million)	29717821658	66.03x
*O. sanctum*_Illumina_Short_insert	73646230 (73.6 million)	7438269230	16.52x
