# Supplementary material for: Unravelling the genome of Holy basil: an “incomparable” “elixir of life” of traditional Indian medicine
Source: BMC Genomics. 2015 May 28;16(1):413. doi: 10.1186/s12864-015-1640-z (PMC4445982; doi:10.1186/s12864-015-1640-z)
Supplement: Additional file 2: — Read length distribution chart for 454 reads. [file 12864_2015_1640_MOESM2_ESM.pdf]

**Additional File 2.** Read length distribution chart for 454 reads

| Range | Number of reads | Percentage of total reads |
|---|---|---|
| 800-999 | 64810 | 10.08 |
| 600-799 | 210235 | 32.69 |
| 400-599 | 137998 | 21.46 |
| 200-399 | 107985 | 16.79 |
| 100-199 | 73890 | 11.49 |
| 99 or less | 48216 | 7.5 |
