# Supplementary material for: Unravelling the genome of Holy basil: an “incomparable” “elixir of life” of traditional Indian medicine
Source: BMC Genomics. 2015 May 28;16(1):413. doi: 10.1186/s12864-015-1640-z (PMC4445982; doi:10.1186/s12864-015-1640-z)
Supplement: Additional file 3: — SOLiD colorspace reads statistics. [file 12864_2015_1640_MOESM3_ESM.pdf]

Additional File 3. SOLiD colorspace reads statistics

**Solid reads orientation**	**F3**	**R3**
file size (csfasta & qual)	8.2 GB & 20 GB	8.2 GB & 20 GB
Maximum Read Length	50	50
Minimum Read Length	50	50
Total Number of Reads	126824255 (126 million)	126824255 (126 million)
Total Number of HQ mate pairs	103221254 (103 million)	103221254 (103 million)
Total Number of Q20 HQ Reads (mate-pair & orphans)	114210103 (114 million)	112773452 (112 million)
Percentage of Q20 HQ Reads	90.05	88.92
Total Number of Bases	6341212750	6341212750
Total Number of Bases in Mb	6341 Mb	6341 Mb
Total Number of Ns	22962009	39215454
Total Number of Ns in Mb	22.9 Mb	39.2 Mb
Percentage of Non-ATGC Characters	0.36	0.62
Number of Reads that contains Ns	6534224	2941993
Percentage of Reads that contains Ns	5.15	2.32
