# Supplementary material for: Unravelling the genome of Holy basil: an “incomparable” “elixir of life” of traditional Indian medicine
Source: BMC Genomics. 2015 May 28;16(1):413. doi: 10.1186/s12864-015-1640-z (PMC4445982; doi:10.1186/s12864-015-1640-z)
Supplement: Additional file 4: — Statistics of transcriptome and whole genome assembly of O. sanctum. [file 12864_2015_1640_MOESM4_ESM.pdf]

Additional File 4. Statistics of transcriptome and whole genome assembly of *O. sanctum*

Description	
Total number of transcripts assembled	69117
Total number of transcripts matching with genome	66891
Number of Transcripts with 100% coverage in whole genome	41079
Number of Transcripts with 90-99% coverage in whole genome	21338
Number of Transcripts with 80-89% coverage in whole genome	2452
Number of Transcripts with 70-79% coverage in whole genome	896
Number of Transcripts with 60-69% coverage in whole genome	474
Number of Transcripts with 50-59% coverage in whole genome	347
Number of Transcripts with less than 50% coverage in whole genome	305
