# Supplementary material for: Unravelling the genome of Holy basil: an “incomparable” “elixir of life” of traditional Indian medicine
Source: BMC Genomics. 2015 May 28;16(1):413. doi: 10.1186/s12864-015-1640-z (PMC4445982; doi:10.1186/s12864-015-1640-z)
Supplement: Additional file 5: — QC statistics of chloroplast de novo assembly at each step. [file 12864_2015_1640_MOESM5_ESM.pdf]

Additional File 5. QC statistics of chloroplast de novo assembly at each step

Particulars	Contigs	Scaffolds	Gapclosed	Gap-closed filtered	Draft genome
Contigs Generated	140	48	48	2	1
Maximum Contig Length	25631	78214	78166	78166	1,42,524
Minimum Contig Length	61	61	61	64356	1,42,524
Median Contig Length	296.5	133.5	70	71261	1,42,524
Total Contigs Length	109671	156442	156070	142522	1,42,524
Total Number of Non-ATGC Characters	55	1373	24	23	25
Percentage of Non-ATGC Characters	0.05	0.88	0.02	0.02	0.018
Contigs >= 100 bp	108	32	32	2	1
Contigs >= 200 bp	98	15	15	2	1
Contigs >= 500 bp	50	9	9	2	1
Contigs >= 1 Kbp	29	5	5	2	1
Contigs >= 10 Kbp	1	2	2	2	1
Contigs >= 1 Mbp	0	0	0	0	0
N50 value	1846	64681	78166	78166	1,42,524
