# Supplementary material for: Unravelling the genome of Holy basil: an “incomparable” “elixir of life” of traditional Indian medicine
Source: BMC Genomics. 2015 May 28;16(1):413. doi: 10.1186/s12864-015-1640-z (PMC4445982; doi:10.1186/s12864-015-1640-z)
Supplement: Additional file 6: — QC statistics of mitochondrial de novo assembly at each step. [file 12864_2015_1640_MOESM6_ESM.pdf]

Additional File 6. QC statistics of mitochondrial de novo assembly at each step

Particulars	Contigs	Scaffolds	Gapclosed	Gap-closed filtered	Draft genome
Contigs Generated	124	41	41	37	37
Maximum Contig Length	16768	43386	43392	43392	43,398
Minimum Contig Length	103	145	145	145	145
Median Contig Length	600	6261	505	524	20029
Total Contigs Length	174923	442572	442370	446661	4,45,881
Total Number of Non-ATGC Characters	0	1616	51	4342	54
Percentage of Non-ATGC Characters	0	0.37	0.01	0.97	0.012
Contigs >= 100 bp	124	41	41	37	37
Contigs >= 200 bp	122	40	40	36	36
Contigs >= 500 bp	68	38	38	34	34
Contigs >= 1 Kbp	45	32	32	29	29
Contigs >= 10 Kbp	1	16	16	16	16
Contigs >= 1 Mbp	0	0	0	0	0
N50 value	3472	24958	24833	25250	25,315
