# Supplementary material for: Unravelling the genome of Holy basil: an “incomparable” “elixir of life” of traditional Indian medicine
Source: BMC Genomics. 2015 May 28;16(1):413. doi: 10.1186/s12864-015-1640-z (PMC4445982; doi:10.1186/s12864-015-1640-z)
Supplement: Additional file 7: — Ocimum sanctum in comparison to Nicotiana tabacum and Solanum lycopersicum. [file 12864_2015_1640_MOESM7_ESM.pdf]

Additional File 7. Genes and intron information of *Ocimum sanctum* in comparison to *Nicotiana tabacum* and *Solanum lycopersicum*

**Intron Length**	***Nicotiana tabacum***		***Solanum lycopersicum***	
	**No. of Introns (*N. tabacum*)**	**No. of Genes (*N. tabacum*)**	**No. of Introns (*S. lycopersicum*)**	**No. of Genes (*S. lycopersicum*)**
5-100	53953	29683	61133	28932
101-200	42520	29001	43646	25714
201-300	17286	14316	18825	14346
301-400	13521	11716	13424	10949
401-500	11789	10325	11396	9524
501-1000	31075	22994	32298	21535
1001-8000	16097	14014	24599	18749
