# Supplementary material for: Unravelling the genome of Holy basil: an “incomparable” “elixir of life” of traditional Indian medicine
Source: BMC Genomics. 2015 May 28;16(1):413. doi: 10.1186/s12864-015-1640-z (PMC4445982; doi:10.1186/s12864-015-1640-z)
Supplement: Additional file 10: — Top 10 Species distribution of hits to UNIPROT database. [file 12864_2015_1640_MOESM10_ESM.pdf]

Additional File 10. Top 10 Species distribution of hits to Uniprot database

**Species**	**No of hits**
Genlisea aurea	30983
Vitis vinifera (Grape)	5082
Fraxinus excelsior (Ash)	1409
Oryza sativa subsp. japonica (Rice)	1325
Solanum tuberosum (Potato)	1287
Orobanche ramosa (Hemp broomrape) (*Phelipanche ramosa*)	941
Medicago truncatula (Barrel medic) (*Medicago tribuloides*)	912
Phelipanche tunetana (Broomrape) (*Orobanche tunetana*)	826
Solanum lycopersicum (Tomato) (*Lycopersicon esculentum*)	712
Prunus persica (Peach) (*Amygdalus persica*)	651
