# Supplementary material for: Unravelling the genome of Holy basil: an “incomparable” “elixir of life” of traditional Indian medicine
Source: BMC Genomics. 2015 May 28;16(1):413. doi: 10.1186/s12864-015-1640-z (PMC4445982; doi:10.1186/s12864-015-1640-z)
Supplement: Additional file 16: — List of 63 protein coding genes of cp genomes used in the phylogenetic analysis. [file 12864_2015_1640_MOESM16_ESM.pdf]

Additional File 16. List of 63 protein coding genes of cp genomes used in the phylogenetic analysis.

S No	List of Genes	S No	List of Genes	S No	List of Genes
1	ndhG	22	petB	43	rbcL
2	ndhH	23	petD	44	rpl14
3	psbJ	24	petG	45	rpl16
4	rpl2	25	petL	46	rpl20
5	rps15	26	petN	47	rpl22
6	rps7	27	psaA	48	rpl32
7	ycf2	28	psaB	49	rpl33
8	atpA	29	psaC	50	rpl36
9	atpB	30	psaI	51	rpoA
10	atpE	31	psaJ	52	rpoB
11	atpF	32	psbA	53	rpoC2
12	atpH	33	psbC	54	rps11
13	atpI	34	psbD	55	rps14
14	matK	35	psbE	56	rps16
15	ndhC	36	psbF	57	rps18
16	ndhD	37	psbH	58	rps2
17	ndhE	38	psbK	59	rps3
18	ndhF	39	psbL	60	rps4
19	ndhI	40	psbM	61	rps8
20	ndhJ	41	psbN	62	ycf3
21	petA	42	psbT	63	ycf4
