# Supplementary material for: Unravelling the genome of Holy basil: an “incomparable” “elixir of life” of traditional Indian medicine
Source: BMC Genomics. 2015 May 28;16(1):413. doi: 10.1186/s12864-015-1640-z (PMC4445982; doi:10.1186/s12864-015-1640-z)
Supplement: Additional file 17: — The list of accession numbers of the chloroplast genome sequences used in this study. [file 12864_2015_1640_MOESM17_ESM.pdf]

Additional File 17. The list of accession numbers of the chloroplast genome sequences used in this study.

No.	Taxon	Family	Order	GenBank Accession number
1	*Salvia miltiorrhiza*	Lamiaceae	Lamiales	JX312195
2	*Ageratina adenophora*	Asteraceae	Asterales	NC_015621
3	*Anthriscus cerefolium*	Apiaceae	Apiales	NC_015113
4	*Daucus carota*	Apiaceae	Apiales	NC_008325
5	*Eleutherococcus senticosus*	Araliaceae	Apiales	NC_016430
6	*Guizotia abyssinica*	Asteraceae	Asterales	NC_010601
7	*Helianthus annuus*	Asteraceae	Asterales	NC_007977
8	*Jacobaea vulgaris*	Asteraceae	Asterales	NC_015543
9	*Lactuca sativa*	Asteraceae	Asterales	NC_007578
10	*Panax ginseng*	Araliaceae	Apiales	NC_006290
11	*Trachelium caeruleum*	Asteraceae	Asterales	NC_010442
12	*Atropa belladonna*	Solanaceae	Solanales	NC_004561
13	*Boea hygrometrica*	Gesneriaceae	Lamiales	NC_016468
14	*Coffea arabica*	Rubiaceae	Gentianales	NC_008535
15	*Datura stramonium*	Solanaceae	Solanales	NC_018117
16	*Ipomoea purpurea*	Convolvulaceae	Solanales	NC_009808
17	*Jasminum nudiflorum*	Oleaceae	Lamiales	NC_008407
18	*Nicotiana sylvestris*	Solanaceae	Solanales	NC_007500
19	*Nicotiana tabacum*	Solanaceae	Solanales	NC_001879
20	*Nicotiana tomentosiformis*	Solanaceae	Solanales	NC_007602
21	*Nicotiana undulata*	Solanaceae	Solanales	NC_016068
22	*Olea europaea*	Oleaceae	Lamiales	NC_013707
23	*Olea europaea subsp. cuspidata*	Oleaceae	Lamiales	NC_015604
24	*Olea europaea subsp. europaea*	Oleaceae	Lamiales	NC_015401
25	*Olea europaea subsp. maroccana*	Oleaceae	Lamiales	NC_015623
26	*Olea woodiana subsp. woodiana*	Oleaceae	Lamiales	NC_015608
27	*Sesamum indicum*	Pedaliaceae	Lamiales	NC_016433
28	*Solanum bulbocastanum*	Solanaceae	Solanales	NC_007943
29	*Solanum lycopersicum*	Solanaceae	Solanales	NC_007898
30	*Solanum tuberosum*	Solanaceae	Solanales	NC_008096
31	*Spinacia oleracea*	Amaranthaceae	Caryophyllales	NC_002202
32	*Arabidopsis thaliana*	Brassicaceae	Brassicales	NC_000932
